# Alcohol-Attributable Death and Burden of Illness among Aboriginal and Non-Aboriginal Populations in Remote Australia, 2014–2018

**DOI:** 10.3390/ijerph20227066

**Published:** 2023-11-15

**Authors:** Renu Unnikrishnan, Yuejen Zhao, Ramakrishna Chondur, Paul Burgess

**Affiliations:** Health Statistics & Informatics, Department of Health, Darwin, NT 0800, Australia

**Keywords:** alcohol-attributable deaths, alcohol-attributable disability-adjusted life years, northern territory, aboriginal health, burden of disease and injury

## Abstract

Harmful use of alcohol is a problem in the Northern Territory (NT), Australia. The aim of this study was to assess and compare alcohol-attributable deaths and the contribution of alcohol to the burden of disease and injury (BOD) among the Aboriginal and non-Aboriginal populations in the NT between 2014 and 2018. The alcohol-use data for adults aged 15+ years old in the NT population was taken from the 2016 National Drug Strategy Household Survey. BOD was measured in disability-adjusted life years (DALY) as part of the NT BOD study. Population-attributable fractions were derived to analyse deaths and BOD. Between 2014 and 2018, 673 Aboriginal and 392 non-Aboriginal people died of harmful use of alcohol, accounting for 26.3% and 12.9% of the total deaths in the Aboriginal and non-Aboriginal population, respectively. Alcohol caused 38,596 and 15,433 DALY (19.9% and 10.2% of the total), respectively, in the NT Aboriginal and non-Aboriginal population for the same period. The alcohol-attributable DALY rate in the Aboriginal population was 10,444.6 per 100,000 persons, six times the non-Aboriginal rate. This study highlights the urgent need to reduce harmful alcohol use in the NT, which disproportionately affects Aboriginal peoples in rural and remote areas.

## 1. Introduction

Alcohol is one of the most widely abused substances globally, and harmful consumption levels impact a range of social and health conditions [1]. In 2016, an estimated three million (5.3%) deaths were alcohol-attributable, and the harmful use of alcohol was responsible for 5.1% of the global burden of disease and injury (BOD). Globally, alcohol was the seventh leading risk factor for deaths and disability in 2016 and the top risk factor among people aged 15–49 years [2]. In Australia, 4.1% of total deaths and 4.5% of the total BOD in 2018 were attributable to alcohol, making it the fifth leading risk factor for BOD [3]. Alcohol use among Australian youth appeared to decline from 6.5% in 2013 to 5.9% in 2016; however, there was no change in the rates of risky drinking [4]. The most likely age group to drink at risky levels were males in their 40s (29%), with males twice as likely as females to drink at risky levels [4]. In 2018, alcohol use was the second largest contributor to total BOD among Aboriginal and Torres Strait Islander peoples (hereafter respectfully referred to as Aboriginal), accounting for 10.5% of the total BOD in Aboriginal Australians [5]. The alcohol-attributable mortality rate was five times higher for the Aboriginal compared to the non-Aboriginal population during the period from 2013 to 2017 [6].

In the Northern Territory (NT) of Australia, 30% of the population was identified as Aboriginal in 2016 [7]. The NT has a high burden of chronic diseases [8] and poor healthcare access, and most of the areas of the NT are geographically remote [9]. The NT population’s age-adjusted DALY rate or BOD per person was 77% higher than the total Australian population, and the NT Aboriginal population experienced 3.6 times more BOD per person than the NT non-Aboriginal population [8]. The NT has a younger population and a slightly higher proportion of male residents [10]. Aboriginal people are less likely to drink alcohol than other Australians; however, for those who do drink, risky drinking is very high in the Aboriginal population [11]. The NT has the highest per capita alcohol consumption in Australia and the highest rates of alcohol-related violence and crime [12]. In 2016, the per capita alcohol consumption was 11.9 L per person in the NT, 27% higher than the national average [13,14]. The NT has the highest number of alcohol-related admissions to the emergency department and intensive care units in Australia [15,16]. Furthermore, the NT also had the highest rate of alcohol-induced (wholly alcohol-related) mortality compared to other states and territories in Australia, with 16.7 deaths per 100,000 persons in 2017 [6]. The alcohol-attributable mortality rate in the NT was 3.5 times the Australian mortality rate, and the rates in Aboriginal peoples were 9–10 times higher than the national rate between 2004 and 2006 [17]. There is a paucity of NT studies on alcohol-attributable deaths (*AAD*) and alcohol-attributable BOD by demographic characteristics to quantify the impact of alcohol misuse and to understand the underlying issues for the NT.

The aim of this study is to assess *AAD* and alcohol-attributable disability-adjusted life years (*AADALY*) among the Aboriginal and non-Aboriginal populations in the NT.

## 2. Materials and Methods

Cause of death data for NT residents was extracted from the Australian Coordinating Registry [18], which included all registered deaths of NT residents across Australia, together with the coded causes of death and demographic information of the deceased. BOD was calculated using *DALY*, calculated as the sum of the years of life lost (YLL) due to premature mortality and the years lived with disability caused by disease and injury [19]. The *DALY* data were taken from the recent NT BOD study (2014–2018) [8]. The estimated resident population stratified by age, sex and Aboriginal status was used as denominators for the rates of *AAD* and *AADALY* [20]. Age-specific alcohol consumption prevalence for adults aged 15+ years old was obtained from the 2016 National Drug Strategy Household Survey (NDSHS) [4], and the consumption prevalence in the NT population was estimated separately by age, sex and Aboriginal status. The 2016 NDSHS reported alcohol consumption using the National Health and Medical Research Council (NHMRC) 2009 guidelines [4]: “standard drink” (10 g of alcohol/12.5 mL of pure alcohol) was used for measuring the alcohol consumption. Australia’s national alcohol guidelines recommend no more than two standard drinks per day or 10 standard drinks a week to reduce the health risks over a lifetime [21]. The revised Australian alcohol guidelines by the NHMRC, however, do not describe a risk-free level of drinking [21,22].

Population attributable fraction (*PAF*) for alcohol was used to determine the proportion of harm attributable to alcohol [23]. Most comparative risk assessments use an indirect method to calculate *PAF* [24]. In this method, the *PAF* by age and sex for each condition was calculated using prevalence of alcohol consumption and relative risk (*RR*) of alcohol in causing individual conditions [23]. Seven categories of alcohol consumption were chosen consistent with the Global Burden of Disease Study (GBD study) (0, 12 g/day (g/d), 24, 36, 48, 60 and 72 g/d) and 10-year age groups for people aged 15+ years [25]. GBD Study measures mortality and morbidity from major illnesses, injuries and risk factors for different countries, territories and selected subnational locations [25].

The list of 30 common diseases/health conditions attributable to alcohol misuse resulting in deaths and *DALY* and the *RR* were taken from the GBD study 2019 [25]. The *RR* was assumed to be identical for the Aboriginal and non-Aboriginal populations. Some conditions within the *International Statistical Classification of Diseases and Related Health Problems 10th Edition* (ICD-10), such as alcohol cardiomyopathy, are wholly attributable to alcohol, and the alcohol *PAF* in these cases is considered to be 100% [23] (listed in Supplementary Table S1). Some other conditions are partially attributable to alcohol with a *PAF* less than 100%. In this study, we consider underlying (e.g., alcoholic liver cirrhosis and alcohol poisoning) and nine multiple (e.g., motor vehicle accident where a person recorded a high blood–alcohol concentration) causes of death to identify wholly alcohol-related (wholly alcohol-attributable) deaths where death is a direct consequence of alcohol. The partially *AAD* are the deaths that have strong associations with alcohol misuse (e.g., mouth and oesophageal cancers); however, there was no reference to alcohol misuse or contribution on the death certificate/cause of death [6].

In this study, some of the wholly alcohol-related diseases come under subcategories of 30 diseases or conditions. They were not isolated from the main disease category, as *RR* was only estimated for the main disease categories [23]. Although these conditions are wholly alcohol-related (which means their *PAF* is 100%), if we calculate *PAF* for these conditions under the main disease category using Levin’s formula [24], the risk will be underestimated. To provide an alternate method to minimise the risk of underestimation of the population burden of alcohol-related mortality and morbidity, in this study, we have calculated the wholly alcohol-related deaths separately. The *PAF* for partially alcohol-attributable conditions was calculated by using the dataset with wholly alcohol-related conditions excluded from the total conditions or the main disease category. The wholly alcohol-related deaths and *DALY* were calculated separately and then deducted from the total deaths and *DALY* to estimate the partially alcohol-attributable mortality and *DALY*. As there were no individual-level data for *DALY*, the wholly alcohol-related *DALY* was estimated on the proportion of alcohol-related YLL and total YLL. The wholly alcohol-related deaths and *DALY* and the partial *AAD* and *AADALY* were added together to obtain the total *AAD* and *AADALY* [26].

The Levin’s formula used to calculate the *PAF* for an alcohol partially attributable condition where the risk varies by consumption is

$$PAF=\frac{\sum_{c}P_{c}\;\left(RR_{c}-1\right)}{\sum_{c}P_{c}\;\left(RR_{c}-1\right)+1}$$

where *c* is an index for category; $\sum_{c}\;$is the sum of overall risk categories; *P* = prevalence of alcohol use in the population or proportion of the population exposed in each age group, Aboriginal status and sex group [5,24]. The *AAD* and *AADALY* are calculated as

AAD=D⋅PAF and AADALY=DALY⋅PAF

where *D* represents the total number of deaths, and the analyses were conducted using Excel and Stata version 17 [27].

For the conditions with *RR* less than one, we assumed the *RR* to be one because, according to NHMRC advice, there is concern about the evidence of alcohol as protective [21,28], and WHO also recommends that no level of alcohol consumption is safe for our health [29]. Recent studies have determined that alcohol is not protective for coronary artery disease and diabetes and suggest that the protective effect found in earlier studies was overestimated [30,31].

Ethics approval was granted by Human Research Ethics Committee of the Northern Territory Department of Health and Menzies School of Health Research (HREC) (Ref no: HREC-2020-3860).

## 3. Results

There were 5593 deaths in the NT from 2014 to 2018 for all age groups, of which 616 (11%) were wholly alcohol-related deaths and 449 (8%) were partially attributable to alcohol (282 were Aboriginal and 167 non-Aboriginal) (Table 1). The Aboriginal *AAD* represented 26.3% of the total Aboriginal deaths, and the non-Aboriginal *AAD* represented 12.9% of the total non-Aboriginal deaths in the NT. The NT Aboriginal *AAD* rates (182 deaths per 100,000 population annually) and *AADALY* rates (10,444.6 years lost per 100,000 population annually) per NT Aboriginal population were higher than non-Aboriginal NT rates (*AAD* rates 45.7, *AADALY* rates 1798.1 per 100,000). The Aboriginal *AADALY* represented 19.9% of the total Aboriginal *DALY*, and the non-Aboriginal *AADALY* represented 10.2% of the total non-Aboriginal *DALY* in the NT. The NT Aboriginal population experienced 4 times more *AAD* and 5.8 times more *AADALY* than the non-Aboriginal NT population.

**Figure 1:**
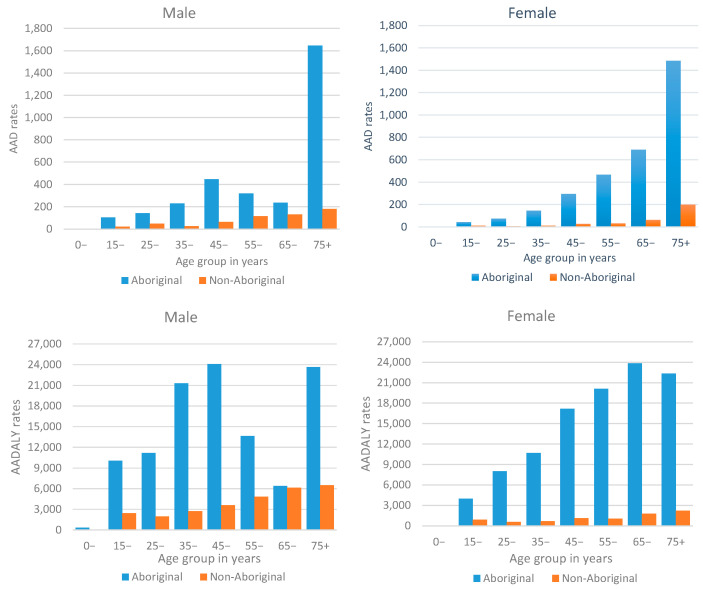
Alcohol-attributable deaths (*AAD*) and disability-adjusted life years (*AADALY*) per 100,000 population by age, sex and Aboriginal status, Northern Territory, Australia, 2014–2018.

Figure 1 illustrates the age distribution for *AAD* and *AADALY* rates by sex and Aboriginal status. Overall, both *AAD* and *AADALY* among the Aboriginal population were higher than in the non-Aboriginal population for all age groups and sex. The difference between Aboriginal and non-Aboriginal *AAD* rates was greatest for age groups 45 years onwards, especially for females. The contrast between Aboriginal and non-Aboriginal *AADALY* rates was greater for females than for males, and among Aboriginal males, a fluctuation of rates by age was observed.

There was a striking similarity in the top three most common causes of *AAD* for Aboriginal and non-Aboriginal NT populations (Table 2, upper panel). Among the Aboriginal and non-Aboriginal population, mouth and pharyngeal cancers (MPC), chronic liver disease (CLD) and suicide were the most common conditions attributable to alcohol misuse resulting in deaths.

Among Aboriginal and non-Aboriginal males, MPC (17.6% and 16.7%, respectively, for Aboriginal and non-Aboriginal), suicide (13.2% and 13.5%) and CLD (10.5% and 13%) were the most common conditions for *AAD* (Supplementary Table S2). For Aboriginal females, CLD (16%), MPC (9.7%) and suicide (6.5%) were the most common conditions, whereas for non-Aboriginal females, CLD (20.5%), suicide (18.1%) and breast cancer (7.8%) topped the list (Supplementary Table S3).

*AADALY* estimates for the NT population by condition, Aboriginal status and sex are compared in Table 2 (lower panel). A total of 38,596 and 15,433 *AADALY* were lost due to alcohol misuse among Aboriginal and non-Aboriginal NT populations during the 5-year study period. The three leading conditions with the highest *AADALY* were homicide and violence, suicide and CLD for the Aboriginal population, and suicide, road traffic injuries and CLD for non-Aboriginal populations (Table 2, lower panel).

The top three leading conditions with the highest *AADALY* were homicide and violence (17%), suicide (13%) and MPC (9%) for Aboriginal males and suicide (15%), road traffic injuries— vehicle occupants (11.3%) and homicide and violence (9.7%) for the non-Aboriginal males (Supplementary Table S4). The top three leading conditions with the highest *AADALY* were homicide and violence (19.3%), CLD (12%) and diabetes mellitus (8.1%) for Aboriginal females. For non-Aboriginal females, the top three leading conditions with the highest *AADALY* were suicide (25.7%), CLD (15.9%) and road traffic injuries—motor vehicle occupants (9.6%) (Supplementary Table S5). *AADALY* and *AAD* proportions were higher among males (two to three times) than females, regardless of Aboriginal status and age group (see Supplementary Tables S2 and S3; Supplementary Tables S4 and S5).

## 4. Discussion

Our findings indicate that *AAD* and BOD are high in the NT, similar to previous findings [23,32]. This is not surprising as the NT has Australia’s highest per capita consumption of alcohol [12]. Gao and others’ study claims that the NT had the highest *AAD* rates and *AADALY* rates in a national study [23]. The *AAD* rate among the NT Aboriginal population in this study was much higher than the Australian Aboriginal *AAD* rate in earlier national studies (9.7%) [33] and NT-based studies (9%) [32]. The higher NT *AAD* proportion (19.0%) in our study compared with the national results may be due to a larger proportion of Aboriginal people (30%) and the unsafe alcohol consumption in the NT compared to Aboriginal people Australia-wide (3.8%) [34]. Secondly, in this study, we calculated wholly alcohol-related deaths separately and added to the partial *AAD*.

*AADALY* among Aboriginal Australians as a whole in 2018 was 10.5% of the total Aboriginal BOD [5], which was less than the NT Aboriginal *AADALY* proportion (19.9%) in this study. The difference in methodological approaches could be the major factor responsible for the differences between this study’s results and previous studies.

The burden of alcohol’s harm in this NT study was higher than previously reported [35]. AIHW reports that in 2011, the proportion of the total *DALY* attributable to alcohol use in the NT was 8.5% [35], less than observed in this study. According to Gao and others, *AAD* in the NT in 2010 was 116, and *AADALY* was 2091, while nationally, it was 5555 and 188,538, respectively [23]. The *AAD* in the NT in Gao and others’ study represented 11.8% of the NT total deaths and 17.6% of the NT total *DALY* [23], in which the *AAD* rate was lower, but *AADALY* rates were higher than our study results (15.6%). We found that *AAD* and *AADALY* rates were substantially higher in males than in females, regardless of ethnicity, which was consistent with AIHW findings [3] and other studies [32].

In this study, the alcohol-attributable mortality and morbidity in the Aboriginal population were higher than in the non-Aboriginal population for all age groups. High alcohol consumption in the Aboriginal population has been associated with socioeconomic disadvantage, remoteness and intergenerational trauma [36,37]. The past and current inequalities, racism and higher levels of emotional and social distress contribute to higher alcohol consumption and associated harm [37]. In this study, MPC is the leading alcohol-attributable condition resulting in death and the fifth leading alcohol-attributable condition resulting in burden. This could be due to MPC being a combination of three categories of cancer: lip and oral cavity, nasopharyngeal and other cancers in the oral cavity and pharynx.

Some of the deaths partially attributed to alcohol misuse could have a great influence on other contributors. However, some studies have shown the neurotoxic, hepatotoxic and carcinogenic properties of alcohol make it a potent risk factor for BOD [38], and alcohol consumption has been associated with an increased risk of cardiovascular diseases [39], stroke [39], kidney diseases [40], suicide [41], breast cancer [42] and diabetes [43].

Previously, researchers considered the protective effects of low alcohol intake on coronary heart disease (CHD), diabetes mellitus and stroke; however, the evidence for such a protective effect is increasingly being questioned [2,30,44]. The recent Mendelian randomisation and meta-analyses studies reported that alcohol use leads to health loss across populations regardless of the amount [2], and the protective effect or risk reduction is an artefact of epidemiological methods and large datasets [2,45,46,47]. The revised Australian alcohol guidelines by the NHMRC do not describe a risk-free level of drinking [22], and according to the global BOD study 2016, no amount of alcohol is safe [2,29]. In this study, we assume there is no protective effect of alcohol consumption in terms of CHD and diabetes prevention, contributing to an increase in *AAD* and *AADALY* in this study.

There are limitations to the methods used to calculate *PAF*s, as this relies on the accuracy of the population’s self-reported alcohol consumption estimates and the availability and quality of the *RR* estimates. Aboriginal-specific *RR* for alcohol-related conditions is unavailable due to a scarcity of studies among Aboriginal people. Therefore, the *RR* was taken from the global BOD study [25]. The same *RR* has been used throughout all the age groups and for both *AAD* and *AADALY*. Applying the same relative risk for specific countries to calculate the *PAF* will likely result in biases due to differences in drinking habits and interactions between alcohol consumption and other risk factors that are unique to each country (such as the degree of poverty) [48,49]. To improve comparative risk analysis methodologies, more country-specific research is necessary [24,49].

The *AADALY* was proportionally estimated using YLL. The lack of individual-level data in the calculation can cause errors that happen when the group’s attributes are assigned to an individual. As the number of deaths does not directly calculate *AADALY*, it is likely that *AADALY* was under- or overestimated and not identical to *AAD*. The other limitation of this study was the lack of good-quality prevalence data. There are missing values for alcohol consumption data from the NDSHS, and the data had to be smoothened for the analysis. The NDSHS did not collect data from homeless people and remote Aboriginal communities, which was a significant limitation for the NT.

Even though the 2019 NDSHS report is a more updated and advanced report of alcohol consumption data than the 2016 report, we have used the 2016 NDSHS report to match the NTBOD study time period. We have used the direct method to estimate the prevalence of alcohol consumption, collected data from the NDSHS and we have not included the per capita estimates. NDSHS used self-reported data, which had likely underestimated the true alcohol consumption data, and these data were then used to calculate indirect estimates of *PAF*s. Excessive drinking can sometimes be underreported in surveys because of recall, social desirability and nonresponse bias. The deaths due to alcohol-related conditions could sometimes be incorrectly identified, especially in rural areas.

However, this study provides recent and updated estimated *AAD* and *AADALY*, which are useful to consider in the context where alcohol consumption rates are high. Considering NHMRC guidelines, the negative *RR* values have been removed from the study, thus excluding all the protective effects from this model. The wholly alcohol-related deaths and *DALY* were calculated separately and then deducted from the total deaths and *DALY* to estimate the partially *AAD* and *AADALY*. The indirect method was used for calculating *PAF*, which allowed the *PAF* to be fitted to match the drinking prevalence of the population of interest.

Alcohol-attributable mortality and BOD are much higher (4 times and 5.8 times, respectively) in the Aboriginal population compared to non-Aboriginal people. Culturally appropriate interventions that focus on younger generations, policy measures that could reduce total population-level alcohol consumption, supply reduction, supporting/increasing Aboriginal participation in alcohol policy/licensing, reintroduction of banned drinker register and improvements to the socioeconomic status of the disadvantaged population are some aspects needed to reduce the substantial health loss attributable to alcohol in Aboriginal and non-Aboriginal populations in the NT [12,50]. Further research is required in this area.

## 5. Conclusions

The harmful use of alcohol contributes to the high population mortality and morbidity in the NT. In our study, we have applied a new method to minimise the risk of underestimation of alcohol-related harm and update the NT estimates for the burden of alcohol-related harm. This provides policy-makers with a more accurate estimation of the burden of illness and a method with which to track progress in the long-term reduction of alcohol-related harm.

## Figures and Tables

**Table 1:** Alcohol-attributable deaths (*AAD*) and alcohol-attributable disability-adjusted life years (*AADALY*), Aboriginal and non-Aboriginal population, Northern Territory, Australia, 2014–2018.

	Aboriginal	Non-Aboriginal	Total
Total deaths	2560	3033	5593
Wholly alcohol-related deaths (%)	391 (15.3%)	225 (7.4%)	616 (11%)
Partially *AAD* (%)	282 (11%)	167 (5.5%)	449 (8%)
Total *AAD* (%)	673 (26.3%)	392 (12.9%)	1065 (19.0%)
*AAD* rates per 100,000 population	182.0	45.7	86.7
Total *DALY*	194,427	151,618	346,044
Wholly alcohol-related *DALY* (%)	26,125 (13.4%)	10,288 (6.8%)	36,413 (10.5%)
Partially *AADALY* (%)	12,471 (6.4%)	5145 (3.4%)	17,616 (5.1%)
Total *AADALY* (%)	38,596 (19.9%)	15,433 (10.2%)	54,029 (15.6%)
*AADALY* rates per 100,000 population	10,444.6	1798.1	4400.4

Note: DALY—Disability-adjusted life years.

**Table 2:** Top twenty most frequent alcohol-attributable deaths (*AAD*) and alcohol-attributable disability-adjusted life years (*AADALY*) by Aboriginal and non-Aboriginal population, Northern Territory, Australia, 2014–2018.

Rank	NT Aboriginal			Rank	NT Non-Aboriginal		
	Condition	Deaths	%		Condition	Deaths	**%**
**1**	MPC	97.2	14.4	**1**	MPC	57.4	14.6
**2**	Chronic liver disease	85.6	12.7	**2**	Chronic liver disease	57.1	14.6
**3**	Suicide and SII	70.8	10.5	**3**	Suicide and SII	56.7	14.4
**4**	RTIvehicle occupants	41.1	6.1	**4**	RTI vehicle occupants	23.9	6.1
**5**	RTI other	32.4	4.8	**5**	Stroke	17.0	4.3
**6**	Coronary heart disease	29.3	4.4	**6**	Falls	16.5	4.2
**7**	Homicide and violence	28.5	4.2	**7**	Bowel cancer	13.4	3.4
**8**	Liver cancer	27.8	4.1	**8**	RTI motorcyclists	11.6	3.0
**9**	Stroke	26.1	3.9	**9**	Breast cancer	9.5	2.4
**10**	Poisoning	18.6	2.8	**10**	Coronary heart disease	9.1	2.3
**11**	Diabetes	18.4	2.7	**11**	Poisoning	6.4	1.6
**12**	Breast cancer	14.7	2.2	**12**	Liver cancer	6.4	1.6
**13**	Falls	13.3	2.0	**13**	RTI other	6.3	1.6
**14**	Alcohol use disorders	13.0	1.9	**14**	COPD	6.3	1.6
**15**	Cardiomyopathy	12.0	1.8	**15**	Diabetes	6.0	1.5
**16**	Oesophageal cancer	10.9	1.6	**16**	Alcohol use disorders	6.0	1.5
**17**	Bowel cancer	10.0	1.5	**17**	Other unintentional inj.	5.7	1.4
**18**	Drowning	9.7	1.4	**18**	Other land transport inj.	5.6	1.4
**19**	Chronic kidney disease	9.0	1.3	**19**	Cardiomyopathy	5.2	1.3
**20**	Other unintentional injuries	8.9	1.3	**20**	Oesophageal cancer	3.4	0.9
	Others	95.6	14.2		Others	62.7	16.0
	Total *AAD*	672.9	100		Total *AAD*	392.3	100
		AADALY	%			AADALY	%
**1**	Homicide and violence	6892.4	17.9	**1**	Suicide and SII	2644.3	17.1
**2**	Suicide and SII	4242.6	11.0	**2**	RTI vehicle occupants	1694.9	11.0
**3**	Chronic liver disease	3204.4	8.3	**3**	Chronic liver disease	1616.8	10.5
**4**	RTI vehicle occupants	3005.3	7.8	**4**	Homicide and violence	1349.5	8.7
**5**	MPC	2859.4	7.4	**5**	MPC	1348.1	8.7
**6**	Alcohol use disorders	2351.3	6.1	**6**	Alcohol use disorders	631.4	4.1
**7**	Diabetes	2285.2	5.9	**7**	RTI motorcyclists	453.1	2.9
**8**	RTI other	1543.9	4.0	**8**	Poisoning	435.2	2.8
**9**	Falls	1167.0	3.0	**9**	RTI other	384.3	2.5
**10**	Coronary heart disease	1107.5	2.9	**10**	Bowel cancer	352.8	2.3
**11**	Stroke	899.0	2.3	**11**	Other transport inj.	332.9	2.2
**12**	Epilepsy	813.8	2.1	**12**	Falls	331.6	2.1
**13**	Liver cancer	794.3	2.1	**13**	Liver cancer	327.0	2.1
**14**	Poisoning	761.0	2.0	**14**	Stroke	310.5	2.0
**15**	Chronic kidney disease	643.4	1.7	**15**	Diabetes	289.5	1.9
**16**	Drowning	526.8	1.4	**16**	Drowning	280.4	1.8
**17**	Atrial fibrillation and flutter	516.1	1.3	**17**	Other unintentional inj.	246.7	1.6
**18**	Other unintentional injuries	477.6	1.2	**18**	Epilepsy	216.1	1.4
**19**	Cardiomyopathy	450.2	1.2	**19**	Breast cancer	199.9	1.3
**20**	Other transport injuries	433.5	1.1	**20**	COPD	197.5	1.3
	Others	3621.2	9.4		Others	1791.0	11.6
	Total *AADALY*	38,595.9	100		Total *AADALY*	15,433.4	100

Note: COPD—Chronic pulmonary obstructive disease; Inj—Injuries; NT—Northern Territory; MPC—Mouth and pharyngeal cancer; RTI—Road traffic injuries; SII—Self-inflicted injuries.

## Data Availability

No new data were created or analysed in this study. Data sharing is not applicable to this article.

## Supplementary Materials

The following supporting information can be downloaded at: https://www.mdpi.com/article/10.3390/ijerph20227066/s1, Table S1: Wholly alcohol-related conditions with ICD-10 coding; Table S2: Top twenty most frequent alcohol-attributable deaths in males by Aboriginal and non-Aboriginal population, Northern Territory 2014–2018; Table S3: Top twenty most frequent alcohol-attributable deaths in females by Aboriginal and non-Aboriginal population, Northern Territory 2014–2018; Table S4: Top twenty most frequent alcohol-attributable disability adjusted life years in males by Aboriginal and non-Aboriginal population, Northern Territory 2014–2018; Table S5: Top twenty most frequent alcohol-attributable disability adjusted life years in females by Aboriginal and non-Aboriginal population, Northern Territory 2014–2018.
