# Supplementary material for: Alcohol-Attributable Death and Burden of Illness among Aboriginal and Non-Aboriginal Populations in Remote Australia, 2014–2018
Source: Int J Environ Res Public Health. 2023 Nov 15;20(22):7066. doi: 10.3390/ijerph20227066 (PMC10671330; doi:10.3390/ijerph20227066)
Supplement: Supplementary file 1 [file ijerph-20-07066-s001.zip › ijerph-2651097-supplementary.pdf]

Supplementary Tables

Table S1. Wholly alcohol-related conditions with ICD-10 coding

ICD-10 CODE	Wholly alcohol-related conditions
E244	Alcohol-induced pseudo-Cushing's syndrome
F10	Mental & behavioural disorder due to alcohol
F100	Mental & behavioural disorder due to alcohol use ac intoxication
F101	Mental & behavioural disorder due to harmful alcohol use
F102	Mental & behavioural disorder due to alcohol use dep syndrome
F103	Mental & behavioural disorder due to alcohol use withdrawal state
F104	Mental & behavioural disorder due to alcohol use withdrawal state with delirium
F105	Mental & behavioural disorder due to alcohol use psych disorder
F106	Mental & behavioural disorder due to alcohol use amnesic syndrome
F107	Mental & behavioural disorder due to alcohol Residual and late-onset psychotic disorder
F108	Mental & behavioural disorder due to alcohol other Mental & behavioural disorder
F109	Mental & behavioural disorder due to alcohol Mental & behavioural disorder NOS
G312	Degeneration nervous system due to alcohol
G621	Alcoholic polyneuropathy
G721	Alcoholic myopathy
I426	Alcoholic cardiomyopathy
K292	Alcoholic gastritis
K70	Alcoholic liver disease
K700	Alcoholic fatty liver
K701	Alcoholic hepatitis
K702	Alcoholic fibrosis & sclerosis of liver
K703	Alcoholic cirrhosis of liver
K704	Alcoholic hepatic failure
K709	Alcoholic liver disease unspecified
K860	Alcohol-induced chronic pancreatitis
O354	Maternal care for (suspected) damage to fetus from alcohol
P043	Fetus & newborn affected maternal use alcohol
Q860	Fetal alcohol syndrome (dysmorphic)
R780	Finding of alcohol in blood
T51	Toxic effect of alcohol
T518	Other alcohols
T519	Alcohol unspecified
X45	Accidental poisoning by & exposure to alcohol
X65	Intentional self-poisoning alcohol
Y15	Poisoning alcohol undetermined intent
Y573	Alcohol deterrents adverse effect Rx use
Y90	Evidence of alcohol involvement determined by blood alcohol level
Y900	Blood alcohol level of < 20 mg/100 ml
Y901	Blood alcohol level of 20-39 mg/100 ml
Y902	Blood alcohol level of 40-59 mg/100 ml
Y903	Blood alcohol level of 60-79 mg/100 ml
Y904	Blood alcohol level of 80-99 mg/100 ml
Y905	Blood alcohol level of 100-119 mg/100 ml
Y906	Blood alcohol level of 120-199 mg/100 ml
Y907	Blood alcohol level of 200-239 mg/100 ml
Y908	Blood alcohol level 240 mg/100 ml or more
Y909	Presence alcohol in blood level not spec
Y91	Alcohol involvement determined level intoxication
Y910	Mild alcohol intoxication
Y911	Moderate alcohol intoxication
Y912	Severe alcohol intoxication
Y913	Very severe alcohol intoxication
Y919	Alcohol involvement NOS

Table S2. Top twenty most frequent alcohol-attributable deaths in males by Aboriginal and non-Aboriginal population, Northern Territory 2014–2018

Rank	NT Aboriginal			Rank	NT non-Aboriginal		
	**Condition**	**Deaths**	**%**		**Condition**	**Deaths**	**%**
**1**	MPC	70.9	17.6	**1**	MPC	51.7	16.7
**2**	Suicide and SII	53.2	13.2	**2**	Suicide and SII	41.8	13.5
**3**	Chronic liver disease	42.3	10.5	**3**	Chronic liver disease	40.3	13.0
**4**	RTI motor vehicle occupants	27.9	6.9	**4**	RTI motor vehicle occupants	20.0	6.5
**5**	Coronary heart disease	20.9	5.2	**5**	Stroke	11.8	3.8
**6**	RTI other	19.1	4.8	**6**	Bowel cancer	11.8	3.8
**7**	Homicide and violence	17.2	4.3	**7**	Liver cancer	11.3	3.6
**8**	Liver cancer	13.2	3.3	**8**	Poisoning	9.2	3.0
**9**	Poisoning	12.5	3.1	**9**	RTI motorcyclists	8.1	2.6
**10**	Falls	10.8	2.7	**10**	Oesophageal cancer	7.7	2.5
**11**	Cardiomyopathy	10.0	2.5	**11**	Falls	7.6	2.4
**12**	Oesophageal cancer	9.2	2.3	**12**	COPD	7.0	2.3
**13**	Stroke	9.0	2.2	**13**	Alcohol use disorders	6.0	1.9
**14**	Alcohol use disorders	8.0	2.0	**14**	Diabetes	5.9	1.9
**15**	Drowning	7.7	1.9	**15**	Coronary heart disease	5.8	1.9
**16**	Diabetes	6.3	1.6	**16**	RTI other	5.2	1.7
**17**	Chronic kidney disease	6.0	1.5	**17**	Drowning	5.2	1.7
**18**	Bowel cancer	5.7	1.4	**18**	Cardiomyopathy	5.0	1.6
**19**	Other unintentional injuries	4.7	1.2	**19**	Other unintentional injuries	4.4	1.4
**20**	Epilepsy	4.6	1.2	**20**	Stomach cancer	4.0	1.3
	Others	43.1	10.7		Others	40.4	13.0
	Total AAD	402.4	100.0		Total AAD	310.4	100.0

Note: AAD – Alcohol-attributable deaths; COPD – Chronic pulmonary obstructive disease; NT – Northern Territory; MPC - Mouth and pharyngeal cancer including nasopharyngeal cancer, lip and oral cavity cancer and other oral cavity and pharynx cancers; RTI -- Road traffic injuries; SII – Self-inflicted injuries

Table S3. Top twenty most frequent alcohol-attributable deaths in females by Aboriginal and non-Aboriginal population, Northern Territory 2014–2018

Rank	NT Aboriginal			Rank	NT non-Aboriginal		
	**Condition**	**Deaths**	**%**		**Condition**	**Deaths**	**%**
**1**	Chronic liver disease	43.3	16.0	**1**	Chronic liver disease	16.8	20.5
**2**	MPC	26.3	9.7	**2**	Suicide and SII	14.8	18.1
**3**	Suicide and SII	17.6	6.5	**3**	Breast cancer	6.3	7.8
**4**	Stroke	17.0	6.3	**4**	MPC	5.7	6.9
**5**	Breast cancer	14.7	5.4	**5**	Stroke	5.2	6.4
**6**	Liver cancer	14.6	5.4	**6**	Bowel cancer	4.8	5.8
**7**	RTI other	13.3	4.9	**7**	RTI motor vehicle occupants	3.8	4.6
**8**	RTI motor vehicle occupants	13.2	4.9	**8**	Poisoning	2.4	2.9
**9**	Diabetes	12.0	4.5	**9**	Liver cancer	2.1	2.6
**10**	Homicide and violence	11.3	4.2	**10**	Other land transport injuries	2.1	2.5
**11**	Coronary heart disease	8.5	3.1	**11**	Oesophageal cancer	1.8	2.2
**12**	Lower respiratory infections	6.4	2.4	**12**	Hypertensive heart disease	1.5	1.8
**13**	Poisoning	6.1	2.3	**13**	Falls	1.5	1.8
**14**	Atrial fibrillation and flutter	5.4	2.0	**14**	Other unintentional injuries	1.2	1.5
**15**	Alcohol use disorders	5.0	1.9	**15**	RTI other	1.1	1.4
**16**	Other land transport injuries	5.0	1.9	**16**	Atrial fibrillation and flutter	1.0	1.2
**17**	Hypertensive heart disease	4.8	1.8	**17**	Hepatitis B (acute)	1.0	1.2
**18**	Bowel cancer	4.3	1.6	**18**	Pneumococcal disease	1.0	1.2
**19**	Other unintentional injuries	4.2	1.5	**19**	Cardiomyopathy	1.0	1.2
**20**	Pancreatitis	3.9	1.4	**20**	Other cardiovascular	1.0	1.2
	Others	33.3	12.3		Others	5.7	7.0
	Total AAD	270.2	100.0		Total AAD	81.8	100.0

Note: AAD – Alcohol-attributable deaths NT – Northern Territory; MPC - Mouth and pharyngeal cancer including nasopharyngeal cancer, lip and oral cavity cancer and other oral cavity and pharynx cancers; RTI -- Road traffic injuries; SII – Self-inflicted injuries

Table S4. Top twenty most frequent alcohol-attributable disability adjusted life years in males by Aboriginal and non-Aboriginal population, Northern Territory 2014–2018

Rank	NT Aboriginal			Rank	NT non-Aboriginal		
	Condition	*DALY*	%		Condition	*DALY*	%
**1**	Homicide and violence	4,091.5	17.0	**1**	Suicide and SII	1,864.4	15.0
**2**	Suicide and SII	3,145.6	13.0	**2**	RTI motor vehicle occupants	1,404.2	11.3
**3**	MPC	2,162.1	9.0	**3**	Homicide and violence	1,201.7	9.7
**4**	RTI motor vehicle occupants	2,125.7	8.8	**4**	MPC	1,196.2	9.6
**5**	Alcohol use disorders	1,796.2	7.4	**5**	Chronic liver disease	1,135.2	9.2
**6**	Chronic liver disease	1,470.4	6.1	**6**	Alcohol use disorders	631.4	5.1
**7**	Diabetes	1,119.4	4.6	**7**	RTI motorcyclists	418.6	3.4
**8**	RTI other	920.9	3.8	**8**	Poisoning	354.6	2.9
**9**	Falls	911.0	3.8	**9**	RTI other	325.5	2.6
**10**	Coronary heart disease	811.4	3.4	**10**	Liver cancer	286.6	2.3
**11**	Poisoning	509.5	2.1	**11**	Falls	285.4	2.3
**12**	Epilepsy	493.4	2.0	**12**	Drowning	277.9	2.2
**13**	Drowning	431.3	1.8	**13**	Diabetes	277.7	2.2
**14**	Chronic kidney disease	383.8	1.6	**14**	Bowel cancer	250.4	2.0
**15**	Cardiomyopathy	378.9	1.6	**15**	Other land transport injuries	237.3	1.9
**16**	Stroke	374.0	1.6	**16**	Stroke	237.3	1.9
**17**	Liver cancer	365.0	1.5	**17**	COPD	197.5	1.6
**18**	Other unintentional injuries	289.0	1.2	**18**	Other unintentional injuries	185.5	1.5
**19**	Oesophageal cancer	259.9	1.1	**19**	Coronary heart disease	184.5	1.5
**20**	Bowel cancer	200.2	0.8	**20**	Oesophageal cancer	165.9	1.3
	Others	1,877.1	7.8		Others	1,281.5	10.3
	Total *AADALY*	24,116.4	100.0		Total *AADALY*	12,399.4	100.0

Note: *AADALY* – Alcohol-attributable disability adjusted life years; COPD – Chronic pulmonary obstructive disease; NT – Northern Territory; MPC - Mouth and pharyngeal cancer including nasopharyngeal cancer, lip and oral cavity cancer and other oral cavity and pharynx cancers; RTI – Road traffic injuries; SII – Self-inflicted injuries

Table S5. Top twenty most frequent alcohol-attributable disability adjusted life years in females by Aboriginal and non-Aboriginal population, Northern Territory 2014–2018

Rank	NT Aboriginal			Rank	NT non-Aboriginal		
	Condition	*DALY*	%		Condition	*DALY*	%
**1**	Homicide and violence	2,800.9	19.3	**1**	Suicide and SII	779.9	25.7
**2**	Chronic liver disease	1,733.9	12.0	**2**	Chronic liver disease	481.6	15.9
**3**	Diabetes	1,165.7	8.1	**3**	RTI motor vehicle occupants	290.7	9.6
**4**	Suicide and SII	1,097.0	7.6	**4**	Breast cancer	199.9	6.6
**5**	RTI motor vehicle occupants	879.5	6.1	**5**	MPC	151.9	5.0
**6**	MPC	697.3	4.8	**6**	Homicide and violence	147.8	4.9
**7**	RTI other	623.0	4.3	**7**	Bowel cancer	102.4	3.4
**8**	Alcohol use disorders	555.2	3.8	**8**	Other land transport injuries	95.6	3.1
**9**	Stroke	525.0	3.6	**9**	Poisoning	80.6	2.7
**10**	Liver cancer	429.4	3.0	**10**	Stroke	73.2	2.4
**11**	Breast cancer	350.7	2.4	**11**	Other unintentional injuries	61.1	2.0
**12**	Atrial fibrillation and flutter	342.1	2.4	**12**	RTI other	58.8	1.9
**13**	Epilepsy	320.5	2.2	**13**	Epilepsy	55.6	1.8
**14**	Coronary heart disease	296.1	2.0	**14**	Other blood metabolic disorder	54.5	1.8
**15**	Chronic kidney disease	259.6	1.8	**15**	Falls	46.2	1.5
**16**	Falls	256.0	1.8	**16**	Liver cancer	40.4	1.3
**17**	Poisoning	251.5	1.7	**17**	Fire, burns and scalds	39.5	1.3
**18**	Other land transport injuries	235.9	1.6	**18**	Hepatitis B (acute)	39.0	1.3
**19**	Other unintentional injuries	188.5	1.3	**19**	Pneumococcal disease	39.0	1.3
**20**	Lower respiratory infections	183.5	1.3	**20**	RTI motorcyclists	34.5	1.1
	Others	1,288.2	8.9		Others	162.1	5.3
	Total *AADALY*	14,479.5			Total *AADALY*		
			100.0			3,034.0	100.0

Note: *AADALY* – Alcohol-attributable disability adjusted life years; COPD – Chronic pulmonary obstructive disease; NT – Northern Territory; MPC - Mouth and pharyngeal cancer including nasopharyngeal cancer, lip and oral cavity cancer and other oral cavity and pharynx cancers; RTI – Road traffic injuries; SII – Self-inflicted injuries
